# Leaf Phenological Stages of Winter Oilseed Rape (*Brassica napus* L.) Have Conserved Photosynthetic Efficiencies but Contrasted Intrinsic Water Use Efficiencies at High Light Intensities

**DOI:** 10.3389/fpls.2021.659439

**Published:** 2021-04-15

**Authors:** Younès Dellero, Mathieu Jossier, Alain Bouchereau, Michael Hodges, Laurent Leport

**Affiliations:** ^1^Institute for Genetics, Environment and Plant Protection (IGEPP), National Research Institute for Agriculture, Food and Environment (INRAE), Institut Agro, Université Rennes, Le Rheu, France; ^2^Université Paris-Saclay, NAtional Committee of Scientific Research (CNRS), National Research Institute for Agriculture, Food and Environment (INRAE), Université Evry, Institute of Plant Sciences Paris-Saclay (IPS2), Orsay, France

**Keywords:** *Brassica napus*, senescence (leaf), source sink relationship, water use efficiency, chlorophyll fluorescence, photosynthesis–respiration imbalance, oilseed rape

## Abstract

Leaf senescence in source leaves leads to the active degradation of chloroplast components [photosystems, chlorophylls, ribulose-1,5-bisphosphate carboxylase/oxygenase (Rubisco)] and plays a key role in the efficient remobilization of nutrients toward sink tissues. However, the progression of leaf senescence can differentially modify the photosynthetic properties of source leaves depending on plant species. In this study, the photosynthetic and respiratory properties of four leaf ranks of oilseed rape describing leaf phenological stages having different sink-source activities were analyzed. To achieve this, photosynthetic pigments, total soluble proteins, Rubisco amounts, and the light response of chlorophyll fluorescence parameters coupled to leaf gas exchanges and leaf water content were measured. Photosynthetic CO_2_ assimilation and electron transfer rates, Rubisco and chlorophyll levels per leaf area were gradually decreased between young, mature and senescent leaves but they remained highly correlated at saturating light intensities. However, senescent leaves of oilseed rape had a lower intrinsic water use efficiency compared to young and mature leaves at saturating light intensities that was mainly due to higher stomatal conductance and transpiration rate with respect to stomatal density and net CO_2_ assimilation. The results are in favor of a concerted degradation of chloroplast components but a contrasted regulation of water status between leaves of different phenological stages of winter oilseed rape.

## Introduction

Resource allocation within plant organs, driven by source-sink relationships, is a critical factor determining plant growth capacities and overall productivity for crop species (Smith et al., 2018). Source-sink relationships represent an interesting target for yield improvement and they have undergone a revival of interest recently (Sonnewald and Fernie, 2018). From a functional point of view, a plant can be divided into source organs, corresponding to photosynthetically active tissues exporting photoassimilates (leaves), and sink organs, i.e., parts of the plant where imported photoassimilates are stored or used (seeds and roots for example) (Fernie et al., 2020). Source organs essentially export carbon and nitrogen resources to sink organs through the phloem in the form of sucrose and nitrogen-rich compounds (glutamine, asparagine, ureides, and peptides) (Julius et al., 2017; Tegeder and Masclaux-Daubresse, 2018). However, depending on the phenological stage and the growth conditions, leaves can have both a source and a sink status (import and export of organic carbon and nitrogen) (Chang et al., 2017). This duality concept is mainly inherent to leaf development and aging, in which senescence represents a major nutrient recycling process ultimately leading to cell death and regulated at multiple levels, notably by leaf age, plant life cycle, and environmental conditions (Woo et al., 2019).

Leaf senescence is characterized by the progressive degradation of chloroplasts through the autophagy machinery (chlorophagy), while mitochondria remain active until the final stages of leaf senescence to support ATP production required for carbon and nitrogen export (Keech et al., 2007; Avila-Ospina et al., 2014). Indeed, during the progression of leaf senescence, the active degradation of major components of chloroplasts (chlorophylls, proteins, and lipids) produces metabolic precursors (amino acids, sugars, and fatty acids) that can be either translocated or used as alternative respiratory substrates for mitochondrial metabolism (Chrobok et al., 2016; Barros et al., 2020). Therefore, leaf senescence can strongly influence plant net CO_2_ assimilation, since photosynthesis is progressively inhibited while mitochondrial respiration and associated decarboxylations are strongly stimulated. In parallel, the initiation of leaf senescence induces the degradation of major complexes of the chloroplast electron transfer chain (CETC), notably the reaction centers of photosystems (PS) II and I and their associated antenna (Schottler and Toth, 2014). Such degradations severely reduce linear electron flow through the CETC thus compromising an efficient use of light for the production of ATP and reducing power. Consequently, the activation of photoprotective mechanisms can occur leading to non-photochemical quenching (NPQ), cyclic electron flow and chlororespiration that limit the production of reactive oxygen species (Krieger-Liszkay et al., 2019). However, these mechanisms are dependent of CETC complex ratios that appear to be degraded differentially depending on plant species (Krupinska et al., 2012; Nath et al., 2013; Schottler et al., 2017). Therefore, leaf senescence can also influence the relationship between chloroplastic electron transfer rate and photosynthetic CO_2_ assimilation.

Winter oilseed rape (*Brassica napus* L.) is an oleaginous crop of major importance due to the production of seeds being naturally rich in triglycerides and proteins. Unfortunately, while oilseed rape has a high nitrogen uptake efficiency at the vegetative stage, the crop requires large mineral nitrogen (N) inputs (150–250 kg N/ha) compared with other crops, due to a low N remobilization efficiency at both vegetative and reproductive stages (Malagoli et al., 2005; Bouchet et al., 2016). Therefore, the engineering of source-sink relationships of oilseed rape represents a promising target for maintaining crop seed yield and quality with reduced N inputs by targeting either N remobilization efficiency or sink establishment (Stahl et al., 2019; Dellero, 2020). To date, research on source-sink relationships in oilseed rape have been essentially focused on the analysis of proteolysis mechanisms and water status in either well-watered or stress conditions (Albert et al., 2012; Musse et al., 2013; Gironde et al., 2015; Poret et al., 2016). Notably, young leaves of oilseed rape accumulate protease inhibitors to protect them for protein degradation while old leaves of oilseed rape show enhanced protease activities (aspartic, cysteine, and chloroplastic) (Avice and Etienne, 2014). However, at the metabolic level, other mechanisms could also play an important role in nutrient remobilization from source-to-sink tissues such as the fine regulation of leaf primary metabolism (Dellero et al., 2020a,b). Notably, the regulation of photosynthetic and mitochondrial activities during the progression of leaf senescence in oilseed rape represents an interesting area that has yet to be explored.

In this study, the impact of leaf phenological stages and their inherent sink-source activities for nitrogen metabolism on the photosynthetic and respiratory properties of oilseed rape have been evaluated by characterizing four leaf ranks representing leaf phenological stages with contrasted sink/source balances (L15, L11, L7, and L3). Levels of photosynthetic pigments, proteins and Rubisco and chlorophyll fluorescence parameters coupled to leaf gas exchanges during light-response experiments have been measured. Correlation analysis between net CO_2_ assimilation, photosynthetic pigment contents, Rubisco levels, stomatal conductance and transpiration rate by using different leaf phenological stages showed interesting results.

## Materials and Methods

### Plant Material and Growth Conditions

All experiments were performed on *B. napus* genotype Aviso [“Bracysol” biological resource center (IGEPP)]. Seeds were first incubated for 3 days on soaked blotting paper to allow seed germination and then transferred in 4 L pots filled with a non-fertilized commercial substrate (Falienor, reference 922016F3). Plant growth was achieved in a 6 m^3^ growth chamber with the following climatic conditions: 14 h of light at 22°C and 10 h of dark at 18°C, an ambient air with around 410 μmol CO_2_·mol^−1^ air and a relative humidity of 65–80%, a photosynthetic photon flux density (PPFD) ranging between 100 and 120 μmol photons.m^−2^.s^−1^ following the position of the leaf within the canopy. Plants were irrigated twice a week with a commercial fertilized solution (Liquoplant Bleu, 2.5% N, 5% P, 2.5% K). All experiments were performed on plants grown for 60 days after sowing, possessing 13 leaf ranks (BBCH-19), annotated from the bottom to the top (L3–L15). The two oldest leaves (L1 and L2) had already fallen off after yellowing, confirming that the remobilization processes between sink and source leaves were operating. A first experiment was performed on three different plants to evaluate and select appropriate leaf ranks for further studies (measurement of fresh weight, leaf area and chlorophyll content in SPAD units using limbs and petioles). A second experiment was performed on three different plants to measure specific limb fresh and dry weights and water, photosynthetic pigment, soluble protein, and Rubisco contents. A third experiment was performed on three different plants to measure leaf gas-exchange and PSII fluorescence parameters. The chlorophyll content in SPAD units was also measured for each leaf of each experiment to ensure that the leaves selected for the analyses were at similar stages across all experiments. Some of the data obtained in this study were very similar to other works performed on leaves of the same genotype grown in similar conditions and using analogous split experimental setups (Dellero et al., 2020a,b).

### Leaf Area and Photosynthetic Pigment Contents

Leaves (L15–L3) were harvested by cutting at the base of their mid-vein and used for the measurements of fresh weight and leaf area in the first experiment. Leaf area was measured with the LI-3100C Area Meter (LiCOR, Lincoln, NE, USA). Chlorophyll levels were first approximated in SPAD units using a non-destructive chlorophyll SPAD-502 meter (Minolta) on leaf limbs (10 measurements per leaf). For photosynthetic pigment determinations, five leaf disks (0.8 cm^2^) were punched with a cork-borer in both lamina sides of the leaves and immediately frozen in liquid nitrogen and stored at −80°C. Frozen samples were ground to a fine powder and photosynthetic pigments were extracted in the dark with 400 μL of pure ice-cold acetone. After a 5 min centrifugation step at 12 000 g and 4°C, the supernatant was collected and stored at 4°C in the dark. These steps were repeated three to four times on the pellet with 80% ice-cold acetone until all pigments were extracted (as judged by a fully white pellet). Supernatants were mixed together and 50 μL were diluted in 80% acetone for photosynthetic pigment quantification by spectrophotometry. Chlorophyll a (chl a), chlorophyll b (chl b), carotenoid (carot), and xanthophyll (xant) contents were quantified in μg.mL^−1^ from the measurements of A_663_, A_646_, and A_470_ nm at 25°C as previously described (Lichtenthaler, 1987): chl a = 12.25 A_663_-2.79A_646_; chl b = 21.50 A_646_-5.10A_663_; carot+xant = 5.05A_470_-0.0091chla−0.429chlb.

### Water, Soluble Protein, and Rubisco Contents

Ten leaf disks (0.8 cm^2^) were punched with a cork-borer from both lamina sides of the leaves, frozen in liquid nitrogen and freeze-dried for 72 h. Water content (WC) was calculated from the measurement of the fresh weight (FW, measured directly after harvesting) and the dry weight (DW, measured after freeze-drying) as follow: WC = (FW-DW)/FW. Specific limb fresh and dry weights were calculated from these samples. Freeze-dried samples were ground to a fine powder and soluble proteins were extracted in a buffer containing 20 mM citrate, 160 mM Na_2_HPO_4_ (pH 6.8), a pinch of polyvinylpolypyrrolidone (PVPP) and a tablet of a protease inhibitor mixture (Complete Mini, EDTA-free, Roche) for 50 mL. After a 15 min incubation with orbital shaking at 1500 rpm, samples were centrifuged for 30 min at 12 000 g and 4°C then soluble proteins in the supernatant were quantified using the Bradford reagent and bovine serum albumin as a protein standard. For relative quantification of Rubisco content, 20 μg of soluble proteins were separated by SDS-PAGE (10% acrylamide) and stained with Coomassie Brilliant Blue (Laemmli, 1970). Rubisco large subunit (LSU) content was evaluated with ImageJ using the “Gel analyser” on 8-bit images, as previously described (Dellero et al., 2015).

### Gas Exchange and Chlorophyll Fluorescence Measurements

For these experiments, an entire working day (8–10 h) was required per plant to achieve all measurements on the four leaf ranks considered in this study. Prior to measurements, each plant was transferred to the laboratory bench 1 h after the beginning of the photoperiod. To accommodate any potential side-effects due to different moisture conditions throughout the day, the order of the leaves used for the light response measurements of A_n_ and g_sw_ were modified as follows: L15, L11, L7, L3 for day 1; L3, L7, L15, L11 for day 2 and L11, L3, L15, L7 for day 3. For measurements, each leaf was placed in a gas-exchange chamber (LCF 6400-40, LiCOR, Lincoln, NE, USA) connected to a portable photosynthesis system (LI 6400XT, LiCOR, Lincoln, Nebraska, USA). The following conditions were maintained in the chamber: a leaf temperature of 20–21°C, a 60–70% relative humidity (VPD leaf approximately equal to 0.8), a CO_2_ concentration of 400 μmol CO_2_.mol^−1^ air and an air flow rate of 300 μmol.s^−1^. Chlorophyll fluorescence parameters were measured using default parameters of the leaf fluorescence chamber and calculated as follows (Maxwell and Johnson, 2000): F_v_ = F_m_-F_o_, F_v_' = F_m_'–F_0_', Non Photochemical Quenching (NPQ) = [(F_m_-F_m_')/F_m_'], PSII electron transport rate (J_PSII_) = φ_PSII_×0.5×PPFD×αleaf, with φ_PSII_ corresponding to the quantum yield of PSII [=(F_m_'-F_t_)/F_m_'], PPFD corresponding to the photosynthetic photon flux density in μmol photons.m^−2^.s^−1^ and αleaf to the light absorption coefficient of a leaf [α leaf = 0.85 (Peterson and Havir, 2001)]. Prior to the measurement of F_0_ and F_m_ levels, plants were dark-adapted for 30 min. For light-response curves, plants were adapted 10 min to each PPFD level and infrared gas analysers were “matched” together before each measurement. PPFD levels were successively 0, 25, 50, 100, 200, 300, 400, 500, 750, 1000, 1250, 1500, 1500, 1750, and 2000 μmol photons.m^−2^.s^−1^. Net CO_2_ assimilation rate (A_n_, expressed in μmol CO_2_.m^−2^.s^−1^), stomatal conductance to water vapor (g_sw_, expressed in mol H_2_O.m^−2^.s^−1^), transpiration (Tr, expressed in mmol H_2_O.m^−2^.s^−1^), and intercellular CO_2_ concentration (C_i_, expressed in μmol CO_2_.mol^−1^ air) were calculated from CO_2_ and H_2_O gas exchanges using standard equations described in the LI 6400XT_v6.2 user manual (Part I: The basics/1. System description/Equation summary). The intrinsic water use efficiency (iWUE) was calculated as A_n_/g_sw_ using PPFD values > 0.

### Stomatal Density

Leaves (L15–L3) were harvested by cutting at the base of their mid-vein and three sections of their limbs were randomly selected. The abaxial epidermis of each section was peeled with a fine clamp at the level of secondary veins. Each epidermis was mounted on a glass slide with water and stomatal density of 0.05 mm^2^ sections was measured using a light microscope (Axioskop 2 *plus*, Zeiss) at x40 magnification. The mean value obtained from the three sections of a leaf was used as a biological replicate.

### Statistical Analysis

Means of different leaf ranks were first compared together with a one-way ANOVA followed by a *post-hoc* Tukey's HSD test for multiple pairwise comparisons. Linear correlations between A_2000_, g_sw__2000_, Tr_2000_, C_i__2000_, photosynthetic pigments, protein levels per leaf area and stomatal density were tested with an F-statistic. For each test, a *p*-value < 0.05 was applied. All statistical analyses were carried out using R base v3.5.1 (R Core Team, 2018).

## Results

**Figure 1:**
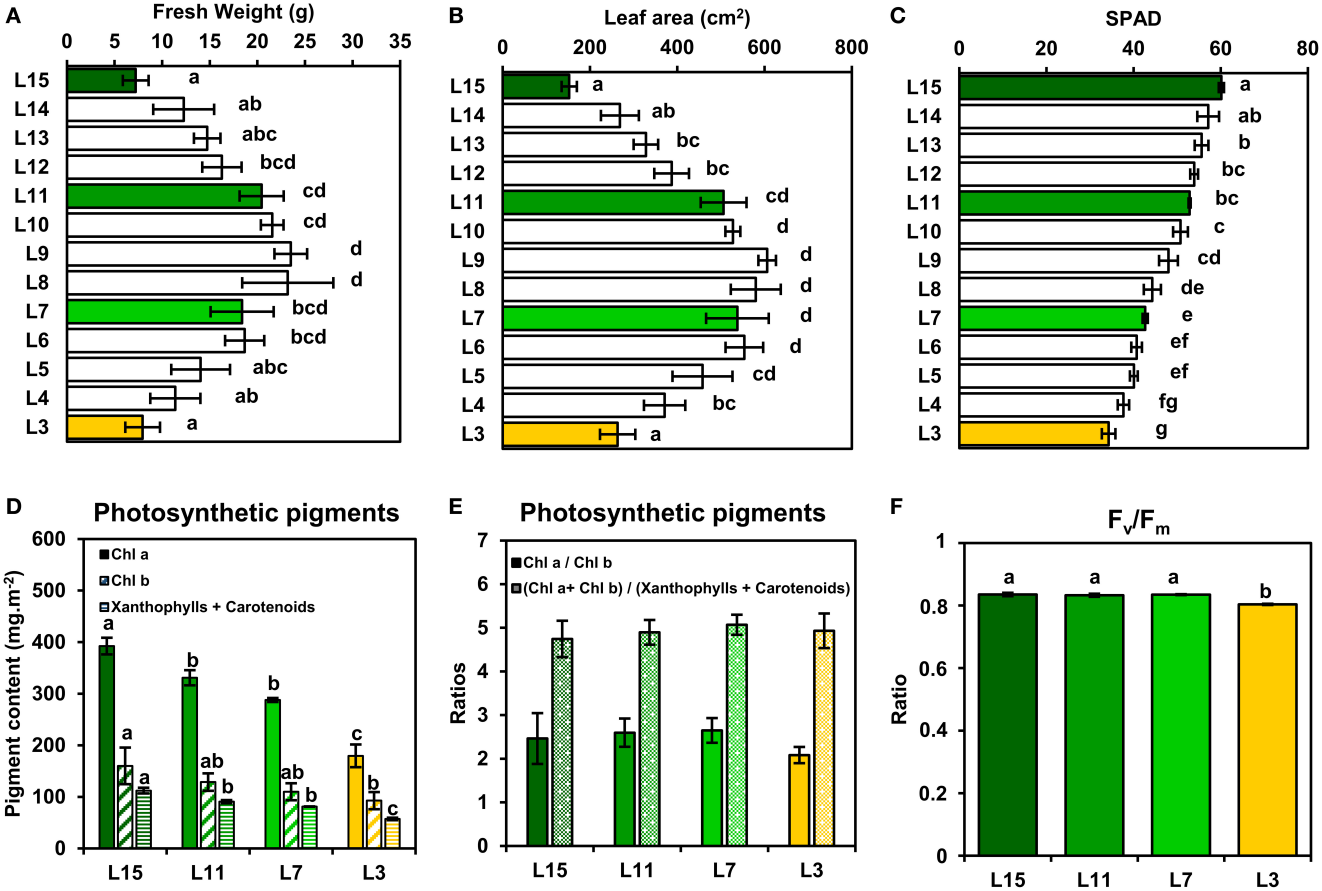
Leaf phenological stages of oilseed rape show a relatively conserved PSII integrity. **(A)** Fresh weight, **(B)** leaf area, and **(C)** chlorophyll content (SPAD units) of oilseed rape leaf ranks. **(D)** Photosynthetic pigment contents and **(E)** ratios and **(F)** PSII maximum quantum yield (F_v_/F_m_) of four leaf ranks as examples of specific leaf phenological stages (L15: young leaf; L11: pre-mature leaf; L7: post-mature leaf; L3: senescent leaf). Values are means ± SD of three independent biological replicates. Different letters indicate groups of mean values that are significantly different between the different leaf ranks (ANOVA-Tukey HSD, *p*-value < 0.05).

In order to evaluate the photosynthetic efficiencies of *B. napus* leaves according to their phenological stage, plants were grown for 2 months at the vegetative stage in growth chambers to reach a 15-leaf stage. At this stage, the two oldest leaves had already fallen off after yellowing, thus confirming the operation of remobilization processes between young, mature and old leaves. Analysis of fresh weight, leaf area and chlorophyll content in SPAD units (Figures 1A–C) allowed to distinguish four leaf ranks representing clearly physiologically differentiated phenological stages. Leaf 15 (L15) corresponded to a young growing leaf, with the highest chlorophyll content and a low leaf area and fresh weight. Leaf 11 (L11) and leaf 7 (L7) corresponded, respectively to fully-expanded mature leaves, with the highest leaf areas and fresh weights but with different levels of chlorophylls. Leaf 3 (L3), with the lowest chlorophyll content, corresponded to an old and senescent leaf which can actively remobilise nutrients to younger leaves (L15 notably), as previously described (Dellero et al., 2020a). Since L11, L7, and L3 leaves showed a gradual decrease of their chlorophyll content in SPAD units compared to L15 that may affect the functioning of their CETC, it was decided to measure the content of various photosynthetic pigments and the maximal quantum yield of PSII (F_v_/F_m_) (Figures 1D–F). All photosynthetic pigment contents (chlorophyll a, chlorophyll b, carotenoids and xanthophylls) were progressively decreased from L15 to L3. Chl a/chl b and (chl a + chl b)/(xanthophylls and carotenoids) ratios were not significantly different between the leaf ranks, with values of around 2.5 and 5, respectively, although the L3 chl a/chl b ratio was slightly lower when compared to the other leaf ranks. Consistently, the maximal quantum yield of PSII (F_v_/F_m_) remained stable between L15, L11, and L7 (values of around 0.835) whereas L3 showed a significantly lower value of around 0.804. Therefore, although chlorophyll content was altered between young, mature and senescent leaves of oilseed rape, the integrity of PSII and the CETC seemed to remain highly conserved.

**Figure 2:**
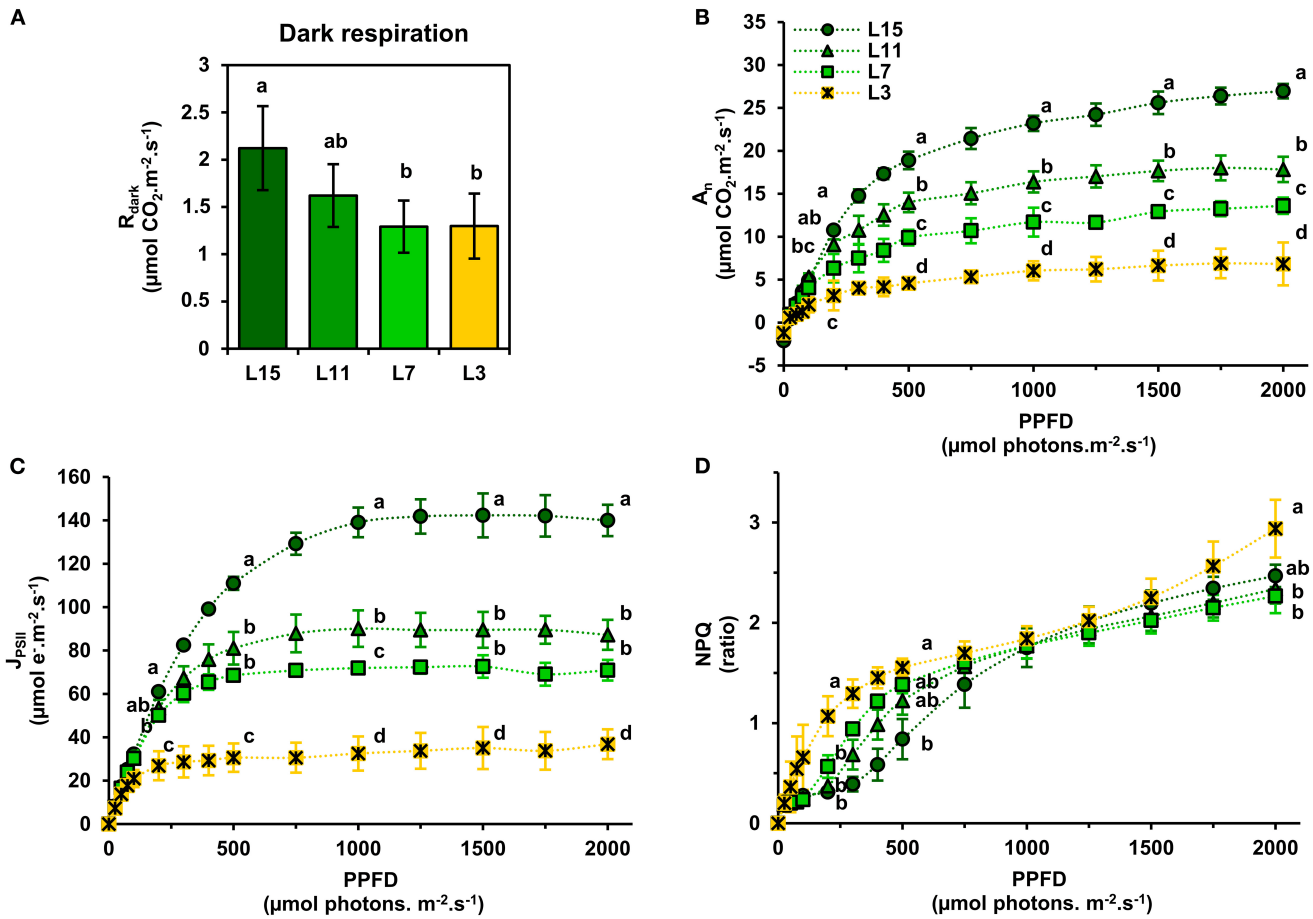
Light-response of net CO_2_ assimilation and chlorophyll fluorescence parameters of oilseed rape leaves at different phenological stages. **(A)** Dark respiration (R_dark_), **(B)** net CO_2_ assimilation rate (A_n_), **(C)** PSII electron transfer rate (J_PSII_), and **(D)** non-photochemical quenching (NPQ). Values are means ± SD of three independent biological replicates. Different letters indicate groups of mean values that are significantly different between the different leaf ranks (ANOVA-Tukey HSD, *p*-value < 0.05) at a given photosynthetic photon flux density (PPFD). L15: young leaf; L11: pre-mature leaf; L7: post-mature leaf; L3: senescent leaf.

**Figure 3:**
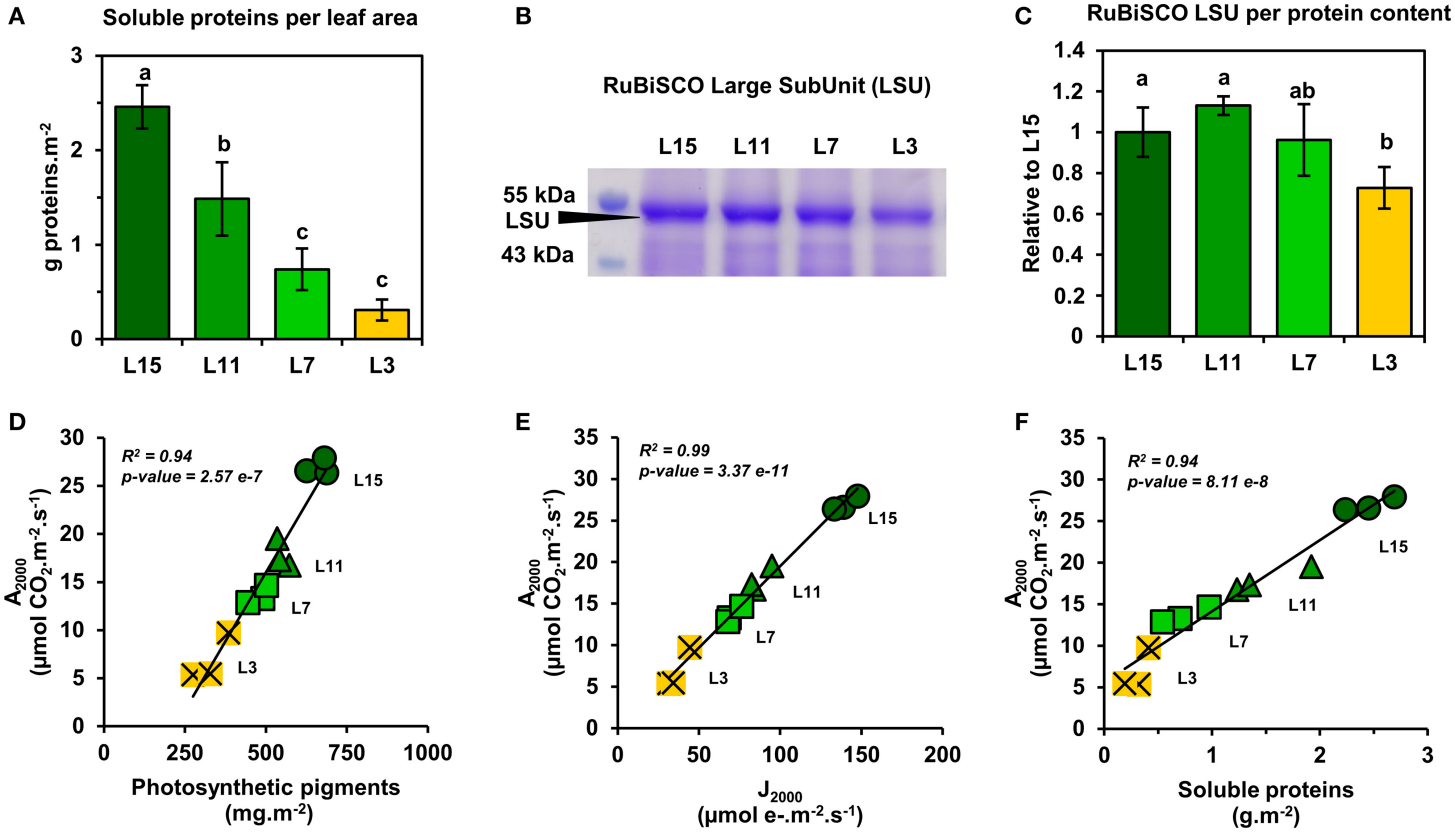
Correlation analysis between the amounts of Rubisco, photosynthetic pigments, J_PSII_, and net CO_2_ assimilation of oilseed rape leaves at different phenological stages and high light intensities. **(A)** Soluble protein contents, **(B)** coomassie blue-stained SDS-PAGE gel showing Rubisco large subunit (LSU), and **(C)** the relative quantification of Rubisco LSU. Correlation analysis at 2000 PPFD between A_n_ and **(D)** photosynthetic pigments, **(E)** J_PSII_, and **(F)** soluble protein contents. Values are means ± SD of three independent biological replicates. Different letters indicate groups of mean values that are significantly different between the different leaf ranks (ANOVA-Tukey HSD, *p*-value < 0.05). L15: young leaf; L11: pre-mature leaf; L7: post-mature leaf; L3: senescent leaf.

Next, it was decided to evaluate further the functioning of the CETC and the photosynthetic CO_2_ assimilation capacity of young, mature and senescent leaves of oilseed rape by performing gas exchange and chlorophyll fluorescence measurements in response to light intensity. We also evaluated dark respiration (R_dark_) as an indicator of mitochondrial respiration. Interestingly, R_dark_ was significantly decreased in L7 and L3 leaf ranks compared to L15 (Figure 2A). Net CO_2_ assimilation rate (A_n_) increased with increasing PPFD for all leaf ranks but it reached a nearly pseudo steady-state level at different PPFD values depending on leaf rank (Figure 2B). Indeed, A_n_ remained relatively stable from 1000 to 2000 PPFD for L11, L7, and L3 and from 1500 to 2000 PPFD for L15. Maximal net CO_2_ assimilation rates at saturating light intensities were significantly different between the different leaf ranks (26.96 μmol CO_2_.m^−2^.s^−1^ for L15, 17.83 for L11, 13.60 for L7 and 6.83 for L3 at 2000 PPFD). Analysis of PSII electron transfer rate (J_PSII_) revealed similar patterns, although it started to reach a pseudo steady-state at an earlier PPFD (Figure 2C). Indeed, J_PSII_ was saturated at around 200 PPFD for L3, 500 PPFD for L7, 750 PPFD for L11, and 1000 PPFD for L15. As was seen with A_n_, maximal J_PSII_ was also significantly different along the different leaf ranks (139.95 μmol e^−^.m^−2^.s^−1^ for L15, 87.24 for L11, 70.90 for L7, and 36.79 for L3 at 2000 PPFD). Since young, mature and old leaves of oilseed rape had relatively conserved ratios of photosynthetic pigments but showed different PPFD thresholds for A_n_ and J_PSII_ saturation, it was decided to measure non-photochemical quenching (NPQ) (Figure 2D). This parameter is an indicator of an activation of photoprotective mechanism allowing the dissipation of excess excitation energy as heat when light energy absorption exceeds light energy utilization (J_PSII_ saturation) and involves the enzymatic conversion of violaxanthin to zeaxanthin (xanthophyll cycle) (Muller et al., 2001). As perhaps expected, there was a significant increase in NPQ at low PPFD levels for senescent L3 ranked leaves that mirrored J_PSII_ saturation compared to young leaves that were not saturated for J_PSII_ (statistically significant at 200 and 500 PPFD). However, between 1000 and 2000 PPFD, NPQ remained similar for all leaf ranks, except for L3 where NPQ became significantly higher at 2000 PPFD compared to L15 and L11 leaves. Overall, these results show a diminution of both photosynthetic CO_2_ assimilation and PSII electron transfer rates from L15 to L3 leaf ranks. Since photosynthetic activity is highly dependent on Rubisco amounts, soluble protein, and Rubisco contents in the young, mature and old leaves of oilseed rape were determined. It was found that soluble protein contents per leaf area gradually decreased from L15 to L3 leaf rank (Figure 3A). Using coomassie blue-stained Rubisco large subunit amounts on SDS-PAGE as a proxy (Figures 3B,C), it could be seen that relative Rubisco amounts per leaf protein content remained rather stable between the leaf ranks, except for L3, which showed a significant decrease of 25% compared to L15 and L11. Overall, soluble protein contents per leaf area seemed to represent a good proxy for Rubisco amounts per leaf area between the different leaf ranks.

**Figure 4:**
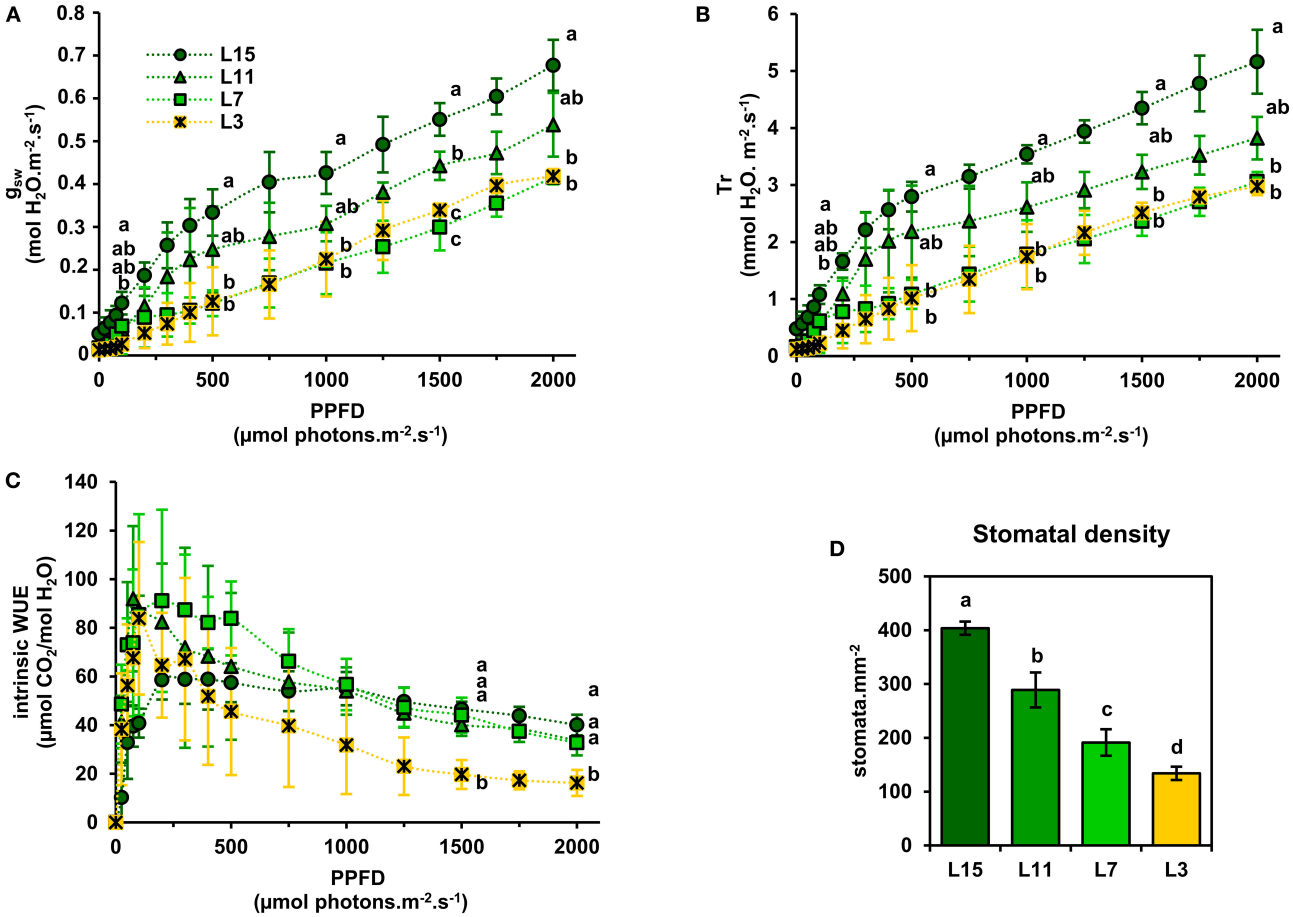
Light-response of stomatal conductance, transpiration, and intrinsic water use efficiency of oilseed rape leaves at different phenological stages. **(A)** Stomatal conductance to water vapor (g_sw_), **(B)** transpiration rate, **(C)** intrinsic water use efficiency (iWUE), and **(D)** stomatal density (abaxial epidermis). iWUE was calculated as A_n_/g_sw_. Values are means ± SD of three independent biological replicates. Different letters indicate groups of mean values that are significantly different between the different leaf ranks for a given PPFD (ANOVA-Tukey HSD, *p*-value < 0.05) at 200, 500, 1000, 1500, and 2000 PPFD. L15: young leaf; L11: pre-mature leaf; L7: post-mature leaf; L3: senescent leaf.

**Figure 5:**
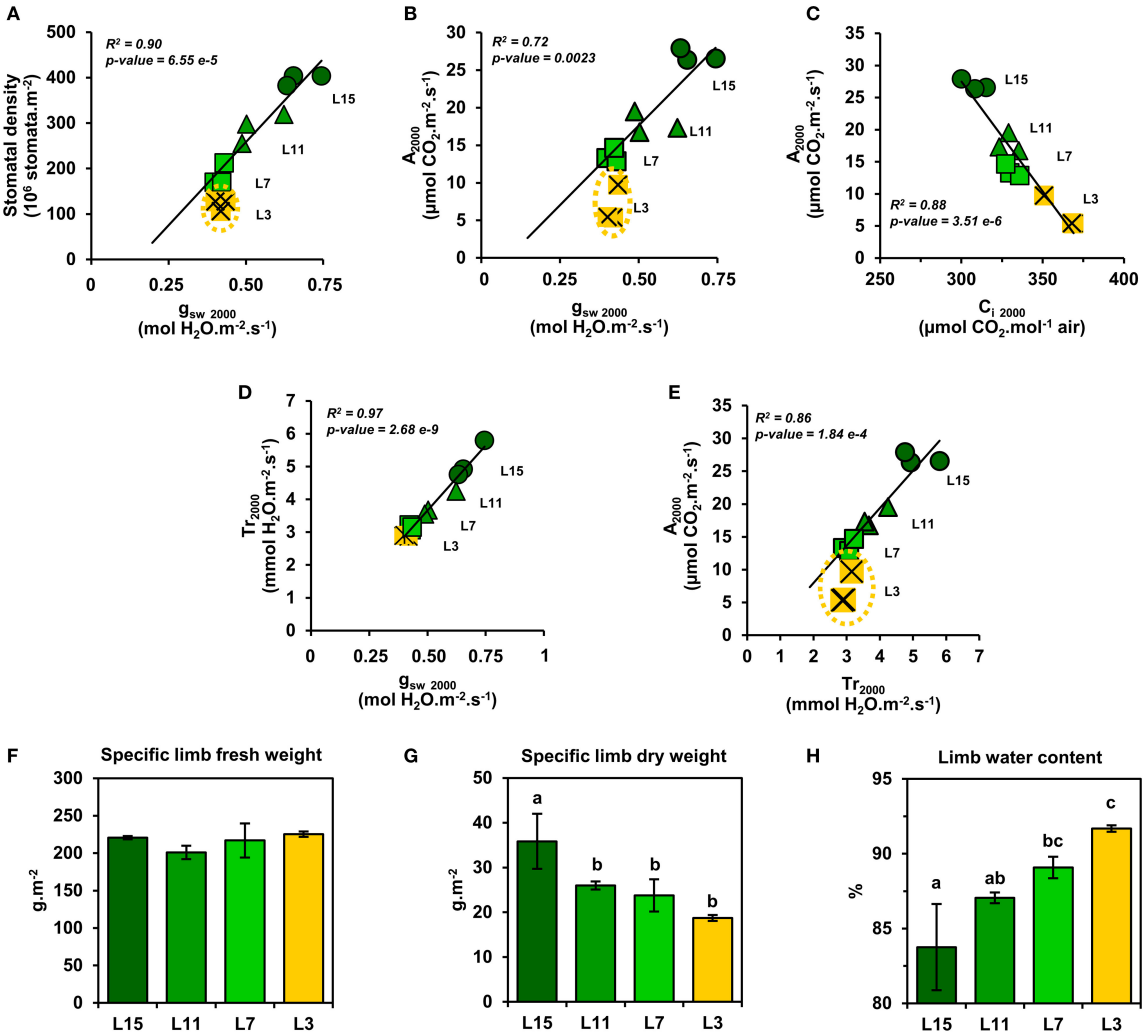
Relationships between stomata and water and CO_2_ exchanges of oilseed rape leaves at different phenological stages and high light intensities. Correlation analysis at 2000 PPFD between: stomatal conductance to water vapor (g_sw_) and **(A)** stomatal density and **(B)** photosynthesis (A_n_); **(C)** photosynthesis (A_n_) and intracellular CO_2_ concentration (C_i_); transpiration and **(D)** stomatal conductance to water vapor (g_sw_) and **(E)** photosynthesis (A_n_). **(F)** Specific limb fresh weight, **(G)** Specific limb dry weight, and **(H)** limb water content. Values are means ± SD of three independent biological replicates. Different letters indicate groups of mean values that are significantly different between the different leaf ranks (ANOVA-Tukey HSD, *p*-value < 0.05). L15: young leaf; L11: pre-mature leaf; L7: post-mature leaf; L3: senescent leaf.

Next, a correlation analysis was performed between A_n_ at 2000 PPFD (A_2000_) and either J_PSII_ at 2000 PPFD (J_2000_), photosynthetic pigments or soluble protein content per leaf area using data obtained from the examined leaf ranks (Figures 3D–F). Interestingly, A_2000_ was highly correlated to J_2000_ (*R*^2^ = 0.99), and also to photosynthetic pigments (*R*^2^ = 0.94) and to soluble protein content (*R*^2^ = 0.94). Since CO_2_ availability to chloroplasts may also participate to the limitation of A_n_ between young, mature and senescent leaves of oilseed rape, the light-response of stomatal conductance to water vapor (g_sw_) and transpiration (Tr) was also examined (Figures 4A,B). Stomatal conductance to water vapor (g_sw_) was constantly increased with increased PPFD but leaf ranks showed significantly different values. L15 showed the highest stomatal conductance, followed by L11 and then L7 and L3. Surprisingly, L7 and L3 leaves exhibited similar stomatal conductance values. The light-response of transpiration (Tr) followed a similar pattern to g_sw_ for the different leaf ranks. Both Tr and g_sw_ did not reach a pseudo steady-state at high light intensities (1500–2000 PPFD) contrary to A_n_ and J_PSII_ and this was observed for all leaf ranks. Consequently, the intrinsic water use efficiency (iWUE, calculated as A_n_/g_sw_) was the highest at low light intensities (around 200 PPFD) and decreased gradually with increasing PPFD levels for all leaf ranks (Figure 4C). However, iWUE was significantly decreased in L3 leaves by up to 50% compared to L15, L11 or L7 leaves at high PPFD values (around 20 vs. 40 μmol CO_2_/mol H_2_O at 1500 and 2000 PPFD). The analysis of stomatal density on the abaxial epidermis revealed that L15 showed the highest stomatal density followed by L11, then L7 and finally L3 (Figure 4D). A correlation analysis between stomatal density, g_sw_, A_n_ at 2000 PPFD showed that there was a certain degree of correlation between stomatal conductance (g_sw2000_) and either stomatal density or photosynthesis (A_2000_) but only for L15, L11, and L7 leaves (*R*^2^ of 0.90 and 0.72, respectively) (Figures 5A,B). Interestingly, senescent leaves (L3) had a higher stomatal conductance compared to their stomatal density thereby suggesting a higher stomatal aperture. However, this was not translated into a higher photosynthetic rate compared to the other leaf ranks (Figure 5B). Since changes in stomatal aperture can affect the diffusion of CO_2_ within intercellular spaces and subsequently photosynthesis, we also analyzed the intercellular CO_2_ concentration at 2000 PPFD (C_i__2000_) (Figure 5C). C_i__2000_ values were gradually increased from L15 to L3 leaves but C_i__2000_ was negatively correlated with A_2000_ for all leaf ranks (*R*^2^ = 0.88). Considering leaf transpiration rate, we found a high correlation with stomatal conductance between all leaf ranks (*R*^2^ = 0.97) and a higher value in the senescent L3 leaves compared to their photosynthetic activity (Figures 5D,E). Finally, we analyzed the specific limb dry and fresh weights of young, mature and senescent leaves of oilseed rape. We found that limb water content increased significantly from L15 to L3, and this was associated with a decrease of dry weight per leaf area, while fresh weight per leaf area remained unchanged (Figures 5F–H).

## Discussion

Source-to-sink remobilization of nutrients is a major process determining plant growth and productivity. Leaf senescence, which leads to the active degradation of chloroplast components (chlorophylls, photosystems, Rubisco) in source leaves while maintaining mitochondrial energy production, plays a key role for the efficient reallocation of carbon and nitrogen to sink tissues. However, the timing of leaf senescence and the kinetics of chloroplast component degradation will modify the photosynthetic properties of source leaves and their net CO_2_ assimilation rates (balance between photosynthesis, photorespiration, and mitochondrial respiration). In oilseed rape, source-to-sink nutrient remobilization processes can operate between different leaf phenological stages, when plants are grown at the vegetative stage notably (Clement et al., 2018; Dellero et al., 2020a). Therefore, we took advantage of this to study the impact of leaf phenological stages and their inherent sink-source activities on the photosynthetic and respiratory properties of oilseed rape.

In this study, we found that PSII functioning remained relatively stable between young, mature and senescent leaves, while a gradual decrease of soluble proteins (including Rubisco) and chlorophyll levels was observed. These phenological stages also showed conserved ratios for photosynthetic pigments and PSII fluorescence properties (similar F_v_/F_m_ values and an activation of NPQ at light intensities saturating J_PSII_) (Figures 1, 3). Since the light intensity used for plant growth in our study (100–120 μmol photons.m^−2^.s^−1^) is low when compared to natural daylight (up to 2000 μmol photons.m^−2^.s^−1^), it is possible that our growth conditions may have prevented an increase of photoinhibition in senescent leaves that could occur under natural daylight conditions. However, in Arabidopsis plants grown under light intensities similar to those used in our work (100–120 μmol photons.m^−2^.s^−1^), the analysis of leaf phenological stages revealed that chlorophyll levels decreased faster than protein levels during natural senescence and this was associated with a decrease of chl a/chl b ratio and PSII maximal quantum yield efficiency (Nath et al., 2013; Tamary et al., 2019). Interestingly, the PSII reaction centers (D1 protein) were rapidly degraded while antennas from PSII and PSI and PSI reaction centers remained conserved (Nath et al., 2013). Since PSII and PSI reaction centers only harbor chl a while PSII and PSI antennas harbor both chl a and chl b (Caffarri et al., 2014), our results suggest that contrary to Arabidopsis, the sequential dismantling of chloroplast antennas and reaction centers in oilseed rape may proceed through a concerted manner. This hypothesis was also supported by the activation of NPQ mechanisms at light intensities saturating J_PSII_ in young, mature and senescent leaves (Figures 2C,D). Indeed, NPQ is triggered by the ΔpH across the thylakoid membrane and it mainly occurs within LHCII and PSII core and requires a functional PsbS protein and the xanthophyll-zeaxanthin cycle (Nicol et al., 2019). Therefore, the activation of NPQ in all of the studied phenological stages suggests a conservation of LHCII and PSII core proteins during chloroplast degradation.

Our results showed that young, mature and senescent leaves of oilseed rape share a similar maximal photosynthetic activity with respect to their photosynthetic capacities (chlorophyll, Rubisco, CETC) at high light intensities (Figure 3). Considering the leaf phenological stages studied and their different metabolic states (balance between growth and nutrient remobilization), a conserved correlation between net CO_2_ assimilation, chlorophyll levels and Rubisco content was not necessarily expected. Indeed, net CO_2_ assimilation rates have been modeled previously as a trade-off between photosynthesis (Rubisco carboxylation steps), photorespiration (CO_2_ released by the glycine decarboxylase complex and controlled by Rubisco oxygenation steps) and mitochondrial respiration (CO_2_ released by the tricarboxylic acid cycle) (Farquhar et al., 1980). Previous studies reported the central role of mitochondrial metabolism during senescence processes in Arabidopsis (Keech et al., 2007; Chrobok et al., 2016). Particularly, the catabolism of many amino acids is activated to supply the tricarboxylic acid cycle for mitochondrial energy production until late stages of senescence (Hildebrandt et al., 2015; Dellero, 2020). In addition, day mitochondrial ATP production in source leaves can be up to four times higher at high light intensities (1500 PPFD) compared to low light intensities (200 PPFD) (Shameer et al., 2019). Therefore, an increase of mitochondrial respiration at high light unbalancing net CO_2_ assimilation with respect to chlorophyll and/or protein content could have been expected in mature and senescent leaves. However, whether amino acid catabolism represents a major flux for mitochondrial respiration in mature and senescent leaves of oilseed rape is still a matter for debate (Dellero et al., 2020a,b). Moreover, boosting mitochondrial respiration may in return have a positive effect on photosynthesis. Indeed, CO_2_ released by pyruvate dehydrogenase, isocitrate dehydrogenase and the GABA shunt can significantly diffuse toward chloroplasts and subsequently increase chloroplastic CO_2_ concentration (Tcherkez et al., 2017). In addition, we have shown that dark respiration (essentially reflecting mitochondrial respiration) was significantly reduced in mature and senescent leaves of oilseed rape (Figure 2A). Although dark respiration still represents 1.3 μmol CO_2_.m^−2^.s^−1^ in L3 leaves (almost 20% of their net CO_2_ assimilation), the inhibition of glycolysis and mitochondrial metabolism in the light would lower its overall impact on net CO_2_ assimilation rate (Tcherkez et al., 2009). Nevertheless, the ratio for light/dark inhibition of mitochondrial respiration during senescence remains to be investigated in plants.

**Figure 6:**
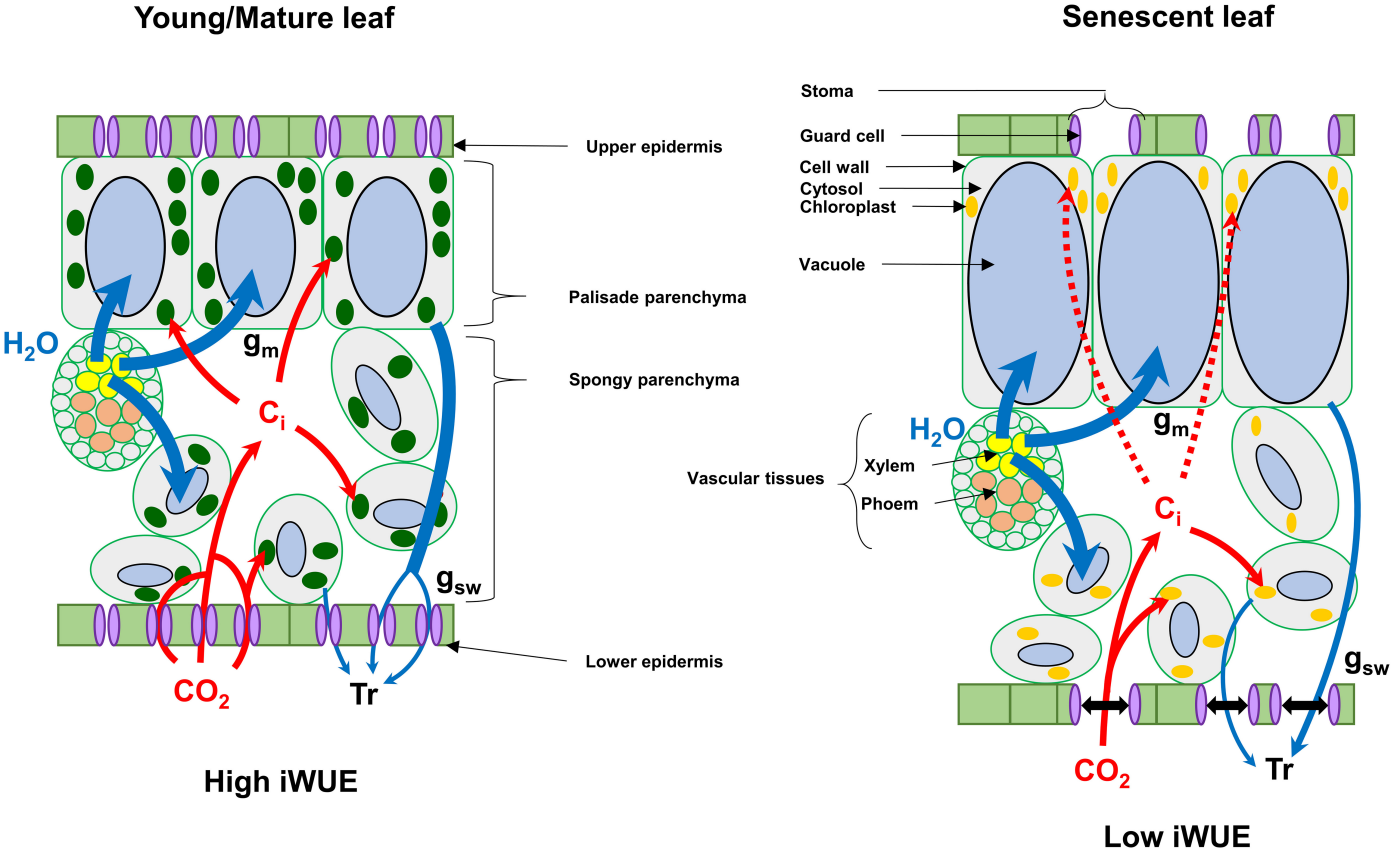
Proposed model for the regulation of intrinsic water use efficiency (iWUE) by senescence in winter oilseed rape leaves at high light intensities. Leaf senescence in winter oilseed rape leads to an increase of the vacuolar volume of cells of the palisade parenchyma, which can limit photosynthesis by decreasing mesophyll conductance of CO_2_ (Musse et al., 2013; Sorin et al., 2015; Lu et al., 2019; Hu et al., 2020). In this model, senescent leaves of oilseed rape increase their stomatal aperture compared to young/mature leaves to increase the diffusion of CO_2_ toward chloroplasts for photosynthesis. C_i_, intercellular CO_2_ concentration; Tr, Transpiration; g_m_, mesophyll conductance; g_sw_, stomatal conductance to water vapor.

We found that senescent leaves of oilseed rape had a lower iWUE compared to the other leaf phenological stages, which was mainly due to a higher transpiration rate (Tr) and stomatal aperture (stomatal density/g_sw_) with respect to the net CO_2_ assimilation levels (Figures 4, 5). Previous studies on winter oilseed rape using low-field proton nuclear magnetic resonance demonstrated that leaf senescence re-orchestrated limb structures and intracellular water flux (Musse et al., 2013; Sorin et al., 2015). Interestingly, the size and the vacuolar volume of cells of the palisade parenchyma increased significantly in mature and senescent leaves while cells of the spongy parenchyma remained weakly affected. These morphological changes were also associated with a gradual increase of the leaf water content and a gradual decrease of leaf dry weight between young, mature and senescent leaves. In our study, we found similar results for leaf water content (Figures 5F–H) but the differences of iWUE between L3 and L15/L11/L7 were not specifically correlated with the gradual increase of leaf water content, thereby suggesting that the lower iWUE at saturating PPFD in L3 leaves was not specifically associated to the regulation of leaf water status. Whether this water influx within mature and senescent leaves of oilseed rape has an important role for the adjustment of leaf osmotic potential or the export of nutrients from source-to-sink tissues remains an open question (Musse et al., 2013; Sorin et al., 2015). Another hypothesis for the increase of stomatal conductance with respect to photosynthesis in senescent leaves involves the regulation of chloroplastic CO_2_ concentration (C_c_) by mesophyll conductance. Our results have shown that net CO_2_ assimilation was negatively correlated with intracellular CO_2_ concentration (C_i_) between young, mature and senescent leaves of oilseed rape (Figure 5C), thereby suggesting that CO_2_ accumulates within intercellular spaces of the leaf when it is not assimilated by Rubisco. However, the Rubisco activity is mainly driven by chloroplastic CO_2_ concentration which depends essentially on stomatal aperture and the capacity of intercellular CO_2_ to diffuse through cell walls, plasma membranes and chloroplast envelopes (mesophyll conductance) (Gago et al., 2020). In Arabidopsis and oilseed rape, leaf senescence is accompanied by a reduction of cell wall thickness, which should facilitate CO_2_ diffusion to chloroplasts (Forouzesh et al., 2013; Musse et al., 2013). On the other hand, leaf senescence also increases palisade parenchyma cell size while reducing the volume of chloroplasts (Musse et al., 2013; Chrobok et al., 2016; Tamary et al., 2019). Thus, a longer path within a liquid phase for CO_2_ should affect its diffusion to the chloroplast. Recent studies reported that mesophyll conductance was strongly limited by the cytoplasm in mature leaves of oilseed rape compared to young leaves and significantly contributed to the limitation of net CO_2_ assimilation (Lu et al., 2019; Hu et al., 2020). Therefore, the increase of stomatal aperture in senescent leaves of oilseed rape could be seen as an adaptive strategy to support photosynthesis with respect to senescence-driven changes of leaf anatomy and Rubisco amounts by boosting CO_2_ diffusion toward chloroplasts (Figure 6).

In conclusion, leaf phenological stages of winter oilseed rape (*Brassica napus L*.) have different levels for photosynthetic pigments, Rubisco, maximal PSII electron transfer rate and maximal net CO_2_ assimilation rates under ambient air but the relative ratios between these components remained strongly conserved per unit of leaf area (photosynthetic efficiency), including an efficient NPQ mechanism. Since these photosynthetic efficiencies remained conserved between the different leaf ranks with respect to their photosynthetic capacities, the results are in favor of a concerted degradation of chloroplast components in winter oilseed rape. Besides this, senescent leaves of oilseed rape have a lower intrinsic WUE at high PPFD compared to young and mature leaves, which seems to be an adaptive strategy to regulate photosynthesis with respect to changes of leaf anatomy (Figure 6). An intriguing result that deserves more attention is the gradual decrease of dark respiration between young, mature and senescent leaves. Future work should be focused on the regulation of mitochondrial respiration by the light/dark cycle in young, mature and senescent leaves of oilseed rape at both physiological and metabolic levels to evaluate its contribution to nutrient remobilization processes.

## Data Availability Statement

The original contributions presented in the study are included in the article/Supplementary Material, further inquiries can be directed to the corresponding author.

## Author Contributions

YD: conceptualization, data curation, formal analysis, funding acquisition, project administration, supervision, visualization, and writing—original draft. YD and LL: investigation. YD, MJ, and LL: methodology. AB, YD, MH, MJ, and LL: validation, writing—review and editing. All authors have read and agreed to the published version of the manuscript.

## Conflict of Interest

The authors declare that the research was conducted in the absence of any commercial or financial relationships that could be construed as a potential conflict of interest.
